# A color Doppler ultrasound-guided protocol for the precise placement of nerve catheters: an anatomical validation study in fresh and fixed cadavers

**DOI:** 10.1186/s12871-026-03873-1

**Published:** 2026-05-02

**Authors:** Benedikt Büttner, Holger Rosemann, Nilas Zieseniss, Caspar Mewes, José Hinz, Ashham Mansur, Ingo Bergmann

**Affiliations:** 1 Department of Anesthesiology, Emergency and Intensive Care Medicine, University Medical Center Göttingen, Robert-Koch-Str. 40, Göttingen, Lower Saxony 37099 Germany; 2 Center of Anesthesiology and Intensive Care Medicine, University Medical Center Hamburg-Eppendorf, Hamburg, Germany; 3 Department of Anesthesiology and Intensive Care Medicine, Asklepios Hospitals Schildautal Seesen, Seesen, Germany; 4 Department of Anesthesiology and Intensive Care Medicine, Sana Hospitals Hof, Hof, Germany

**Keywords:** Color Doppler ultrasound, Nerve catheter placement, Regional anesthesia, Cadaver Study

## Abstract

**Background:**

Continuous analgesia via peripheral nerve catheters (PNCs) is highly effective but prone to tip malposition. Direct ultrasound-guided localization of the PNC tip remains challenging, and no universal method is currently established. This cadaver-based study aimed to validate a color Doppler ultrasound-guided protocol for accurate PNC tip placement at the brachial plexus.

**Methods:**

Eleven ultrasound-guided PNC placements were performed (5 fixed/6 fresh cadavers) according to our specific protocol. PNC tip positioning was repeatedly assessed by visualizing bolus injections via color Doppler ultrasound following 0.5 cm retraction if applicable. Good placement was defined as a color Doppler signal directly adjacent to the brachial plexus concomitant with hydrodissection within its fascial compartment. Verification was obtained through anatomical dissection of the PNC tip in situ and evaluation of the maximal staining site after dye injection via the PNC.

**Results:**

The diagnostic agreement between the color Doppler signal and anatomical findings was 100%. In fixed cadavers, correct PNC tip placement was confirmed in 100% (5/5) of the specimens. Among the fresh cadavers, 83.3% (5/6) were correctly placed. One secondary malposition was detected by ultrasound and confirmed macroscopically. Qualitative analysis of dye staining revealed good localization in 81.8%, acceptable localization in 9.1%, and poor localization in 9.1% of the samples.

**Conclusion:**

Our standardized color Doppler ultrasound-guided protocol reliably facilitates precise PNC tip placement in both fixed and fresh cadavers. Using this method, practitioners can accurately verify and adjust PNC tip position in cadavers. This universally applicable approach may translate to clinical practice but warrants further investigation.

**Trial registration number:**

DRKS00005626

The present validation report was derived from an unpublished study. This study was registered with the German Clinical Trials Register under the clinical trial number DRKS00005626 in July 2015 [Weblink:https://drks.de/search/de/trial/DRKS00005626]

## Introduction

Continuous peripheral nerve blockade is a highly effective analgesic therapy for painful extremity surgeries, offering multiple benefits [1]. Especially at the brachial plexus, incorrect peripheral nerve catheter (PNC) positioning can lead to inadequate or missing analgesia, nerve injury, or unintended anesthesia of adjacent nerves [2]. The reported dislocation rates of interscalene PNCs are as high as 40% [3]. Consequently, accurate positioning of the PNC tip close to the target region is critical for adequate analgesia [2]. Despite various approaches [4–12] and to our knowledge, no standardized protocol allows immediate localization and adjustment of the PNC tip regardless of its type.

Therefore, the aim of our study was to validate a protocol for PNC placement that integrates previous approaches such as hydrolocalization by saline injection, color Doppler ultrasound and gradual retraction. The primary outcome was the ability of the CDS method to accurately identify correct topographical placement of the PNC tip in relation to the dissected anatomical structures. Irrespective of the PNC type, this method should enable reliable localization of the PNC tip in situ and its correction through stepwise PNC retraction.

## Methods

A cadaver-based anatomical pilot study was conducted between August 2015 and January 2017 at the Center of Anatomy at the University Medical Center of Göttingen (Germany). All cadavers were donated with documented consent for scientific and educational use. After approval by the institutional review board of the University Medical Center Göttingen (No. 9/7/13), it was registered with the German Clinical Trials Register under the clinical trial number DRKS00005626 (Weblink: https://drks.de/search/de/trial/DRKS00005626) in July 2015. The present validation report was derived from this unpublished study.

All PNC (Contiplex^®^ D; B. Braun Melsungen AG, Melsungen, Germany) placements were performed by highly experienced senior physicians (IB, BB) under ultrasound guidance (12 MHz transducer, M-Turbo; FUJIFILM SonoSite, Bothell, WA), following a standardized five-step protocol (I-V, see details below). The out-of-plane needle technique and our color Doppler ultrasound-guided protocol were used consistently. In formalin-fixed cadaveric samples, fixation-induced dehydration requires increased gain settings during ultrasound imaging. (I) After the needle tip adjacent to either the interscalene or the costoclavicular brachial plexus was advanced, 10 to 20 mL distilled water was administered to ensure that at least 180° of the plexus circumference was enveloped, allowing sufficient space for PNC advancement. (II) Accordingly, the needle tip was positioned in the respective target region: at the interscalene level, within the scalene groove, lateral to C5/6 of the brachial plexus; at the costoclavicular level, between the brachial plexus cords beneath the clavipectoral fascia. The PNC was then introduced up to 3 cm beyond the needle tip or until slight resistance was encountered. (III) Color Doppler ultrasound-guided localization of the PNC tip was performed by advancing the ultrasound probe 2–3 cm in the direction of the PNC while repetitively injecting 0.5 mL boluses through the PNC and thereby visualizing the characteristic flow-dependent color Doppler signal (CDS) at the PNC tip and the injectate distribution for hydrodissection relative to the plexus. (IV) Malpositions were corrected through stepwise PNC retraction in 0.5 cm increments. (V) After each adjustment, color Doppler-based reassessment was performed using an additional 0.5 mL to confirm repositioning until the CDS indicated a distance < 0.25 cm between the PNC tip and target region. A distance that should remain determinable using the ultrasound equipment employed. The PNC was subsequently fixed by sewing-on. To allow resorption of the initial injectate depot and to close the previously created fluid spaces, 10 mL of very low-concentration (0.005%) methylene blue (MB) dye was injected 24 h after PNC placement. This secondary injection enabled renewed CDS-guided detection of the PNC tip location and its potential dislocation after resorption of the initial depot.

Our standardized protocol was performed in all samples by scanning the target nerve region and surrounding tissue using the *HFL38* ultrasound transducer (13–6 MHz; M-Turbo, FUJIFILM SonoSite, Bothell, WA, USA). Imaging was conducted using the *Nerve* preset with the *medium* flow sensitivity, resulting in a color scale velocity of 15 cm/s and a pulse repetition frequency of 2083 Hz (FUJIFILM SonoSite, Bothell, WA, USA). Good tip positioning was defined by the presence of both a CDS directly adjacent to the brachial plexus and a hydrodissection within the correct fascial compartment. Malposition was assumed when one or more of the following criteria were present: absence of CDS or hydrodissection within the target region; CDS and hydrodissection outside the target region; intraneural CDS or hydrodissection; indeterminate CDS visualization due to acoustic shadowing (e.g., beneath the clavicle), preventing confirmation of PNC tip localization.

This anatomical study was conducted in cadavers to evaluate procedural feasibility, ultrasound-guided PNC positioning, and, in particular, the feasibility of anatomical dissection for in situ determination of the PNC tip under different tissue conditions. Cadavers represent a valuable model for the evaluation of various ultrasound-guided regional anesthesia approaches; [13, 14] however, their respective advantages and limitations differ by cadaver type. Accordingly, the study was conducted in two consecutive phases using both fixed (phase 1) and fresh (phase 2) cadaveric specimens. A total of seven cadavers with average body weights and typical physiques were included. Three cadavers were preserved in formalin and used exclusively in phase 1. Four cadaveric specimens were refrigerated and allocated to phase 2 approximately three to five days post-mortem to maintain realistic tissue elasticity. Owing to postmortem gas accumulation in the tissue, ultrasound-guided PNC placement was not feasible at all interscalene and costoclavicular sites, which led to their exclusion from the study.

In phase 1, five PNC placements were performed in three fixed cadavers at the interscalene or costoclavicular position of the brachial plexus. Formalin preservation increases tissue rigidity and provides structural stability, thereby enabling stepwise anatomical dissection from the skin to the PNC tip without altering its original position. In formalin-fixed cadavers, the in situ position of the PNC tip can thus be determined accurately through anatomical dissection. These cadavers were preserved according to standard institutional protocols via injection into the femoral artery. Cadaveric dissections were performed with the PNC in situ by an experienced anatomist in collaboration with the primary investigator (BB). The brachial plexus was exposed in a layer-by-layer fashion at both the interscalene and costoclavicular levels, while a tissue segment containing the PNC was intentionally preserved along its original course, extending from the skin puncture site to the catheter tip. Dissection proceeded stepwise along this segment toward the tip, thereby preventing accidental catheter displacement. At the interscalene and costoclavicular levels of the brachial plexus, the skin and subcutaneous tissue were removed, and portions of the sternocleidomastoid and the pectoralis major and minor muscles were subsequently reflected. The medial part of the clavicle was carefully resected to improve visualization of the brachial plexus, the carotid artery–jugular vein complex, and the subclavian vessels. Following opening of the prevertebral fascia, the anterior and middle scalene muscles and the brachial plexus sheath within the interscalene space were exposed, while the phrenic nerve was preserved. In fixed cadavers, the rigidity of the fixed tissue enabled precise in situ positioning without displacement during anatomical dissection, allowing direct verification of the PNC tip and implied topographical interpretation of the maximal staining site after methylene blue dye injection (MB maximum). However, altered mechanical properties of their fixed tissue may lead to atypical or impaired PNC navigation. Therefore, in phase 2, six placements were conducted in four fresh cadaveric samples, more closely simulating the handling and dynamics of living tissue. In contrast, the mobility and pliability of fresh cadaveric tissue render in situ visualization of the PNC tip technically difficult in most cases. The site of the MB maximum served as a surrogate marker for the PNC tip position in relation to the brachial plexus and neighboring anatomical structures, primarily based on the authors’ clinical experience and expertise. In all cadaveric specimens, the extent of the MB maximum was assessed as a surrogate for the expected clinical effect of a local anesthetic. These findings were arbitrarily classified as good (MB maximum mainly affecting the intended target region with distinct staining of brachial plexus roots/cords), acceptable (MB maximum within the intended target region with distinct staining of brachial plexus roots/cords), poor (MB maximum outside the intended target region but slight staining of brachial plexus roots/cords) or absent (no staining). Ultrasound measurements (distance from the PNC tip-to-target region) were obtained via integrated ultrasound device tools. Procedural documentation included ultrasound imaging and photographic records of the dissection findings for validation.

The primary outcome was the diagnostic accuracy of the CDS in identifying topographically correct PNC tip placement with respect to dissected anatomical structures. The secondary outcomes included malposition detection rates, agreement with macroscopic dissection findings, PNC tip-to-target region distances and qualitative assessments of MB dye distribution. The results are presented in a descriptive manner. Group differences were tested using the Mann-Whitney U test.

## Results

A total of 11 ultrasound-guided PNC placements and subsequent anatomical dissections were performed in the brachial plexus region of fixed (3 interscalene PNCs/2 costoclavicular PNCs) and fresh (4 interscalene PNCs/2 costoclavicular PNCs) cadaveric specimens. Across all PNC placements, the target region of the brachial plexus was located at a mean depth of 1.70 ± 0.56 cm. No significant difference was observed between interscalene and costoclavicular PNCs (*p* = 0.43). The maximum sonographic depth required to detect PNC malposition during the color Doppler examination was 4.0 cm over all PNC placements. All procedures were technically feasible in accordance with our standardized five-step protocol and yielded evaluable ultrasound and macroscopic data.

**Fig. 1:**
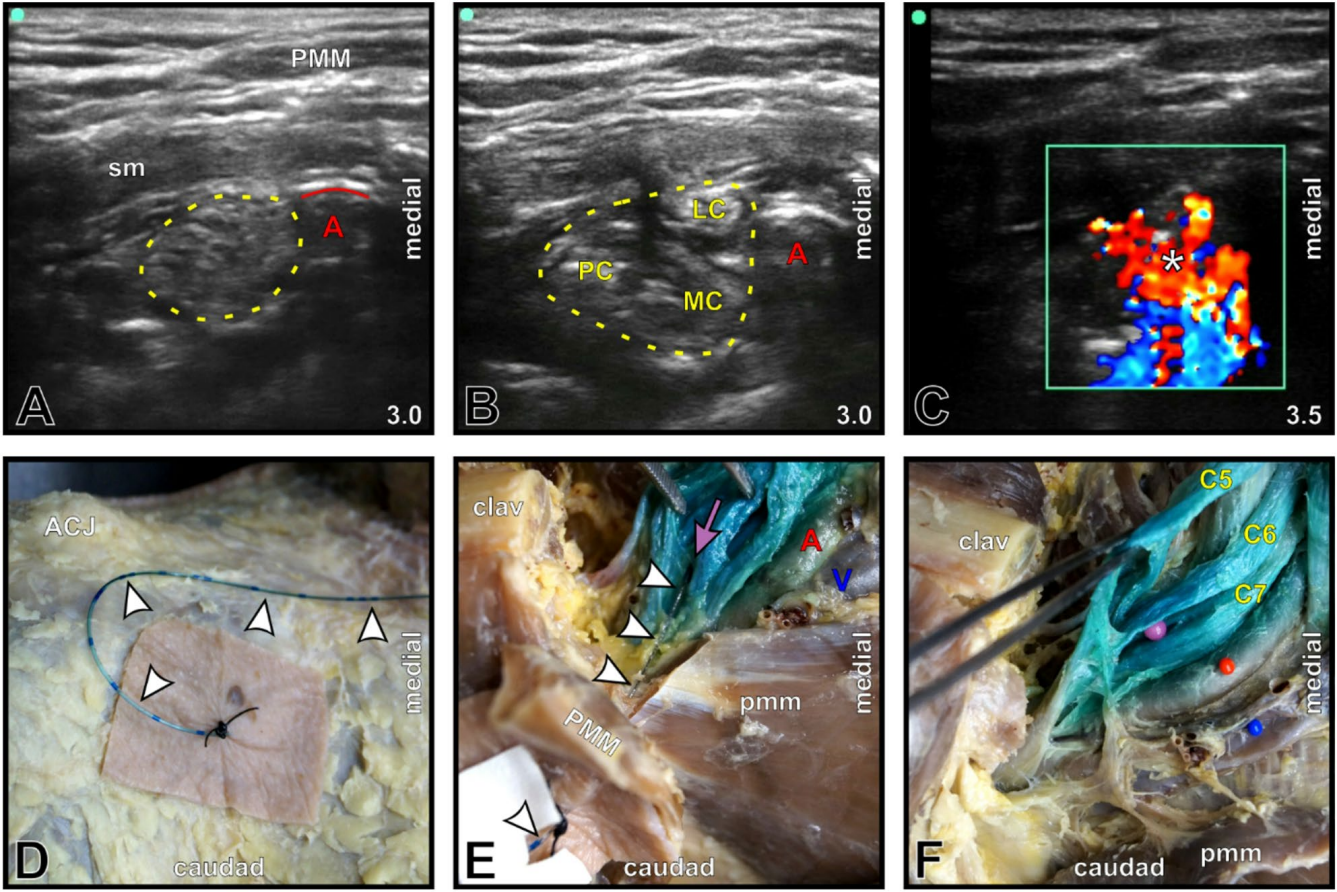
Good PNC tip placement at the costoclavicular brachial plexus in a fixed cadaver. **A** B-mode image showing the costoclavicular brachial plexus (*yellow dotted line*) lateral to the air-filled subclavian artery (**A**). The ultrasound imaging depth [cm] is shown in the lower right corner. **B** Brachial plexus (*yellow dotted line*) following initial depot formation with spread between the lateral (*LC*), medial (*MC*), and posterior (*PC*) cords. *sm*, subclavian muscle. **C** Color Doppler mode image showing a reliable CDS. The *white asterisk* indicates the site of initial hydrodissection and the CDS, corresponding to the good PNC tip position. **D** Stepwise anatomical dissection preserving the sutured PNC (*white arrowheads*) *in situ*, starting from the skin puncture site. ACJ, acromioclavicular joint. **E** Dissection of the PNC tip position (*purple arrow*) within MB-stained brachial plexus cords dorsal to the retracted clavicular part of the pectoralis major muscle (*PMM*) and the resected medial half of the clavicle (*clav*). *pmm*, pectoral minor muscle; V, subclavian vein. **F** MB maximum, separated via forceps, fully corresponding to the PNC tip (*purple pinhead*) and located in the costoclavicular brachial plexus adjacent to the subclavian vessels (*red/blue pinhead*). C5-7, cervical spinal nerves

**Fig. 2:**
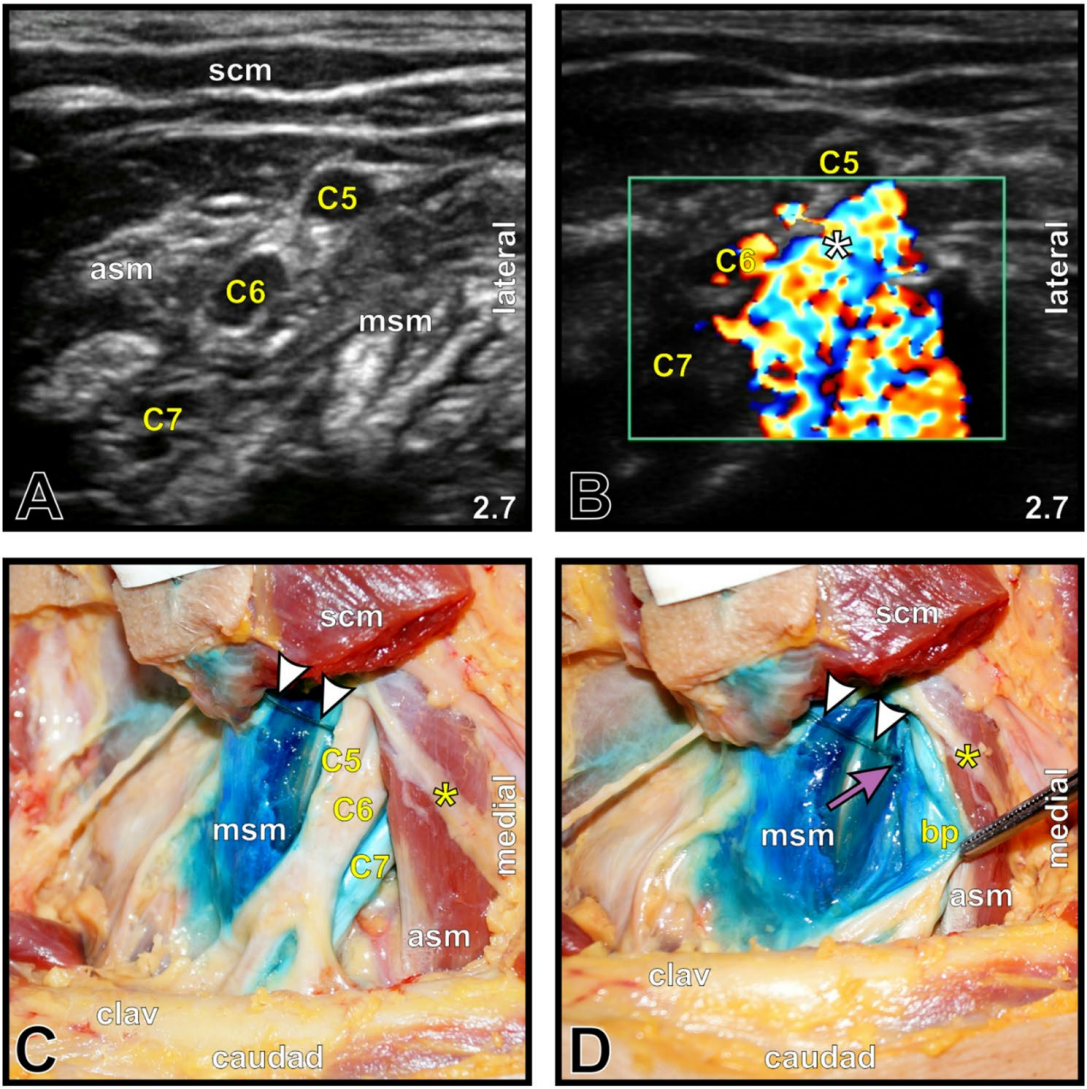
Good PNC tip placement at the interscalene brachial plexus in a fresh cadaver. **A** B-mode image of the cervical spinal nerves (*C5-7*) forming the interscalene brachial plexus between the anterior (*asm*) and middle scalene muscles (*msm*). The ultrasound imaging depth [cm] is shown in the lower right corner. **B** Color Doppler mode image showing a reliable CDS directly adjacent to the interscalene brachial plexus. The *white asterisk* indicates the site of initial hydrodissection and the CDS, corresponding to the good PNC tip position lateral to *C5/6*. **C** Anatomical dissection of the PNC (*white arrowheads*) *in situ*, coursing between the intensely MB-stained middle scalene muscle and the brachial plexus (C5-C7). Note the completely unstained phrenic nerve (*yellow asterisk*) descending caudally over the anterior scalene muscle (*asm*). *clav*, clavicle; *scm*, sternocleidomastoid muscle. **D** Dissection of the PNC tip (*purple arrow*) positioned between the brachial plexus (*bp*), retracted with forceps, and the middle scalene muscle. Note the intense staining of the lateral aspects of the brachial plexus

**Fig. 3:**
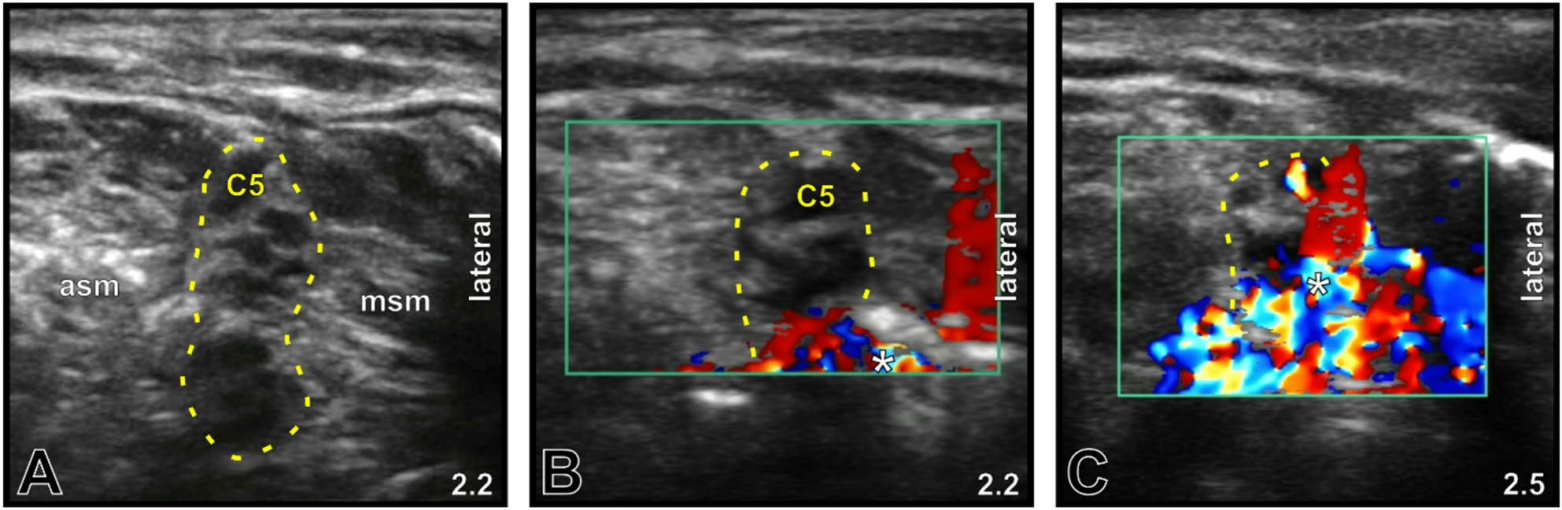
Color Doppler ultrasound-guided PNC tip correction at the interscalene brachial plexus in a fixed cadaver. **A** B-mode image showing the interscalene brachial plexus (*yellow dotted line, C5*) located between the anterior (*asm*) and the middle scalene muscle (*msm*). The ultrasound imaging depth [cm] is shown in the lower right corner. **B** Color Doppler mode image demonstrating an improvable CDS adjacent to the caudal portion of the brachial plexus. The *white asterisk* indicates the site of initial hydrodissection and the CDS, corresponding to the PNC tip position. **C** Color Doppler mode image after PNC retraction by 0.5 cm, showing a distinct and reliable CDS adjacent to the cranial portion of the brachial plexus, which is consistent with optimized PNC tip positioning

**Fig. 4:**
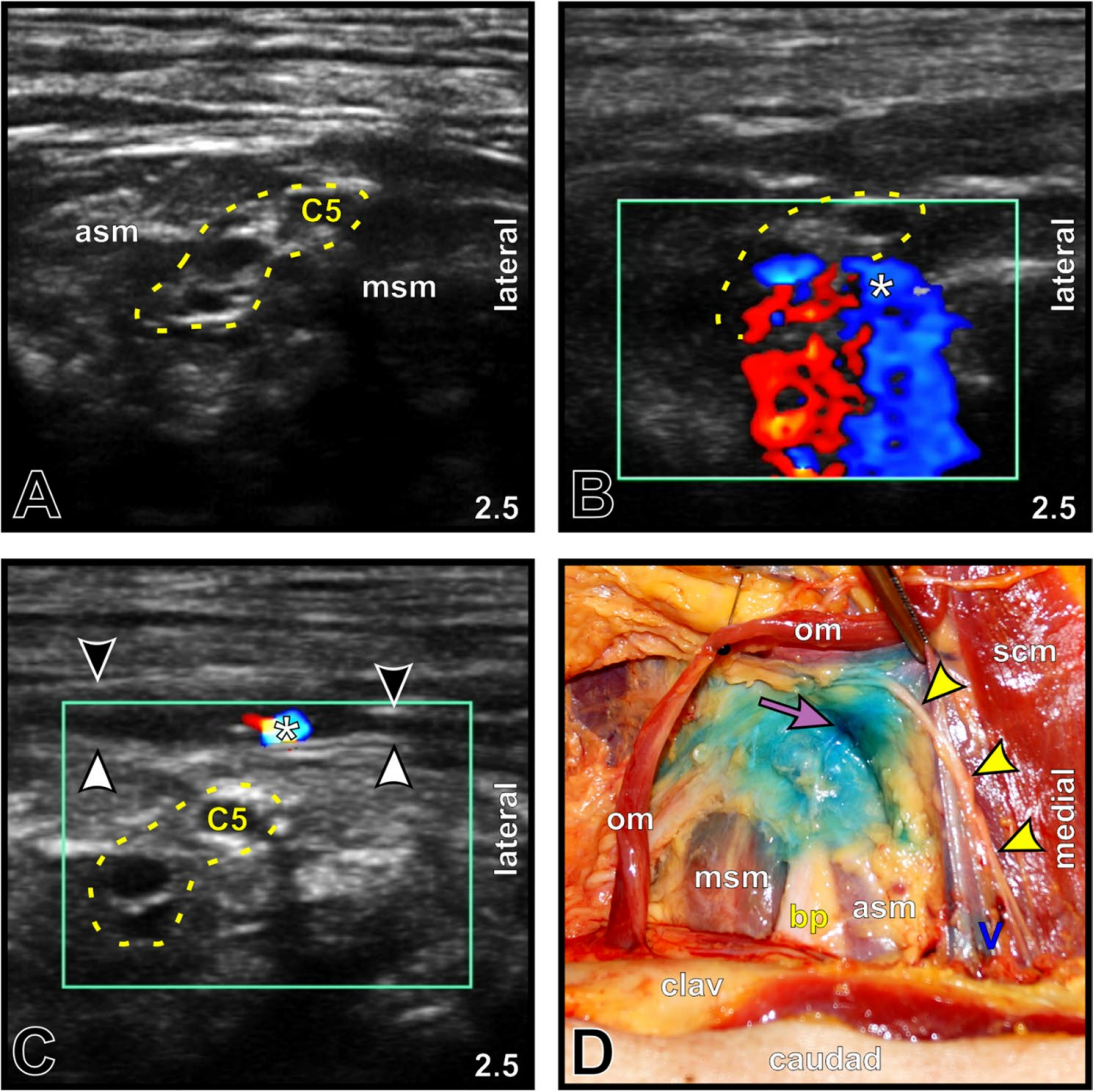
Color Doppler ultrasound-guided detection of a secondary PNC tip malposition at the interscalene brachial plexus in a fresh cadaver. **A** B-mode image of the interscalene brachial plexus (*yellow dotted line, C5*) located between the anterior (*asm*) and middle scalene muscles (*msm*). The ultrasound imaging depth [cm] is shown in the lower right corner. **B** Color Doppler mode image showing reliable CDS during initial PNC placement. The *white asterisk* indicates the site of initial hydrodissection and the CDS, corresponding to the PNC tip position. **C** Color Doppler mode image obtained 24 h later, demonstrating secondary malposition of the PNC tip (*white asterisk*). CDS was located between the superficial (*black arrowheads*) and prevertebral (*white arrowheads*) layers of the cervical fascia. **D** Anatomical dissection confirming malposition of the PNC tip, indicated by the MB maximum (*purple arrow*), situated between layers of the cervical fascia and superior to the unstained brachial plexus (*bp*) within the scalene groove. Note the proximal staining of a descending supraclavicular nerve branch (*yellow arrowheads*) originating from the superficial cervical plexus. *clav*, clavicle; *om*, retracted omohyoid muscle; *scm*, sternocleidomastoid muscle; *V*, internal jugular vein

The CDS-guided protocol allowed topographically correct placement of every PNC tip in fixed cadavers (5/5, 100%; Fig. 1) and in five out of six placements in fresh cadavers (83.3%; Fig. 2), revealing correct placements in 90.9%. The initial insertion depth (mean ± SD) was 5.64 ± 1.58 cm; in 9 of 11 locations (81.8%), PNC withdrawals were performed (Fig. 3), resulting in a final depth of 4.05 ± 0.76 cm. One malposition occurred secondarily in an interscalene PNC of the fresh cadaver group (16.7%), but it was detected after 24 h by ultrasound and subsequently confirmed via macroscopic dissection (Fig. 4).

In addition to binary placement accuracy, the localization of the MB maximum relative to the target region was assessed qualitatively. In the fixed cadavers, the MB maximum was consistently observed directly at the target region in all the cases (Fig. 1). In the fresh cadaver group, the location of the MB maximum relative to the target region was graded as good in four cases (66.7%; Fig. 2), acceptable in one interscalene PNC (16.7%; MB maximum directly beneath the fascia within the middle scalene muscle), and poor in one interscalene PNC (16.7%; MB maximum directly superficial to the prevertebral fascia above the scalene groove) in 6 cases. Across all PNC placements (*n* = 11), the MB maximum was classified as good in nine (81.8%), acceptable in one (9.1%), poor in one (9.1%), and absent in no case.

At initial placement, the ultrasound-measured distance from the PNC tip to the target region was, per protocol, < 0.25 cm across the entire PNC. At 24 h post-placement during MB injection, one secondary malposition was detected, yielding a distance of 0.5 cm between the PNC tip and target region. All ultrasound-determined 24-hour post-placement distances were fully consistent with the PNC tip in situ and/or the location of the MB maximum.

Overall, this yielded complete (100%) diagnostic agreement in both the topographical findings and the distance measurements performed during ultrasound and anatomical dissection in both fixed and fresh samples. No intraneural placements or other complications were observed.

## Discussion

This cadaveric pilot study demonstrated sonoanatomical concordance between the CDS location and the topographical position of the PNC tip relative to the dissected brachial plexus anatomy. Our CDS-guided protocol enables precise placement of the PNC tip through accurate real-time localization and, if needed, direct correction of malposition via iterative retraction and CDS-based reassessment. Furthermore, this CDS-guided approach detected one secondary PNC malposition, which was later also confirmed by dissection.

PNC malposition remains a leading cause of failure of continuous regional anesthesia. Notably, reported rates of PNC dislocation at the interscalene brachial plexus are as high as 40% [3, 5]. However, this evidence is limited, and it largely focuses on secondary dislocation rather than primary misplacement. Previous studies have investigated ultrasound techniques for localizing the PNC tip rather than enabling corrective action. Techniques involving air injection [7] or agitated air-containing fluid [6] can reveal the PNC tip location but inevitably produce acoustic shadowing, precluding further evaluation and preventing ultrasound-guided repositioning. Many practitioners have focused on visualizing the saline injectate itself rather than the PNC tip. This hydrolocalization assesses the “spread of fluid” [5] between anatomical layers, reflecting only the injectate space rather than the true position of the PNC tip. In our opinion, this could be fatally misleading. The space between two fascia hydrodissected by the injectate, such as the initially injected depot along the entire interscalene brachial plexus and the middle scalene muscle, provides a valid “spread of fluid” [5] during saline injection along this plane. However, this hydrodissected space exists only transiently and collapses within minutes once the fluid is resorbed. When this happens, the previously opened fascial pathway from a more caudal directed PNC tip (e.g., at the level of C8/Th1) to the intended target region (e.g., C5/6) closes again, eliminating the intended analgesic effect but providing unwanted anesthesia of neighboring nerve structures. Many device-dependent solutions [8, 15] represent useful alternatives but require proprietary equipment and special skills and are therefore not universally applicable. In contrast, our CDS-guided protocol evaluated in this study can be applied with every PNC type and any color Doppler-capable ultrasound device, providing a low-cost, widely accessible solution. Building on the concept of characteristic flow-dependent CDS real-time visualization in PNC placement, first described by Dhir and Ganapathy as the “medley of colors” [4] and recently verified by Bowens et al. [10], our protocol integrates dynamic perineural flow visualization with hydrolocalization principles [5] and stepwise PNC retraction [11]. This multimodal approach provides direct feedback on fluid dynamics at the PNC tip and enables adjustments until good positioning is achieved. In our cadaveric models, this resulted in good agreement between the CDS findings and the macroscopic anatomy across both the fixed and fresh cadaveric samples. Given the variability in dye spread patterns reported in other cadaveric studies and the resulting inconsistency of evidence [16], only the maximal MB staining site was interpreted as a surrogate marker for the PNC tip position. High topographical accuracy addresses the common clinical challenge of inadequate analgesia due to misplaced PNC [2], which is particularly important in the complex brachial plexus region [17, 18]. The detection of secondary catheter malposition after 24 hours in one fresh specimen highlights a particularly relevant finding: This observation reinforces that CDS-guided visualization of the PNC tip may also be useful as a tool for postoperative bedside assessment of its position after the initial injectate depot has been resorbed, as has already been investigated in patients using the color Doppler technique [19]. This could facilitate decision-making regarding postoperative management in cases of absent or insufficient effect of a PNC procedure – namely, whether the catheter should be removed and replaced, whether an increase in the infusion rate of local anesthetic could be considered, or even whether partial withdrawal of the catheter might be appropriate. From a translational perspective, the ability to verify and correct malpositions in real time represents a substantial advantage over current practices, as PNC malposition is likely the most common cause of inadequate perineural analgesia; PNC can be inserted too deeply into an ineffective plane or dislocated superficially. Furthermore, the method’s independence from specific PNC models or add-on systems may enhance its adaptability in diverse clinical environments, including resource-limited setting techniques for PNC tip localization.

This study has several limitations. Using the out-of-plane needle technique contrasts with most published studies and recommendations, in which the in-plane needle technique is commonly utilized. Although robust evidence is lacking, the authors are convinced that PNC placement scanning the short axis using the out-of-plane technique allows the catheter to be advanced longitudinally along elongated nerves, potentially reducing the risk of dislocation. In contrast, with the in-plane needle technique, the PNC is advanced transversely, perpendicular to the nerve, accompanying the nerve over a much shorter distance and potentially increasing the likelihood of secondary malposition. This belief is primarily based on their own clinical experience of achieving consistently good results. Further studies directly comparing both techniques are required to reinforce the trend hinted in our study. The cadaveric model does not replicate live perfusion, tissue elasticity, or physiological flow conditions, which may affect injectate dynamics. Fixed cadavers, while ideal for anatomical dissection, exhibit altered mechanical properties that influence fluid spread, whereas fresh specimens offer more realistic handling but limited postprocedural verification. Additionally, PNC placements were performed by only two highly experienced senior physicians. The small sample size reflects the exploratory nature of this pilot investigation, in which no blinded evaluation or independent verification of the results was performed. Furthermore, no comparator or placebo arm was included to determine whether the CDS-guided protocol improves the accuracy of PNC tip placement. Functional and clinical outcomes could not be assessed. Therefore, future research should focus on the potential advantages of this approach over conventional PNC placement techniques, as well as on validating these findings in living subjects and assessing both efficacy and safety under clinical conditions.

## Conclusions

To our knowledge, this is the first study to confirm, through anatomical dissection, the true concordance between the CDS and the PNC tip position. In doing so, our CDS-guided protocol for PNC placement builds upon and integrates previous work by other groups [4, 5, 11], offering a unified workflow for both localizing and adjusting the PNC tip. Beyond the experimental setting, this protocol allows the operator to visualize and optimize PNC placement during one intervention and potentially to verify PNC function postoperatively in the ward. This represents a dynamic and universally applicable approach for continuous peripheral nerve blockade, and it could help reduce the frequency of PNC failure. Ongoing and future clinical studies should evaluate its clinical effectiveness and safety, determine interoperator reproducibility, and investigate its impact on patient outcomes such as analgesic success and opioid consumption.

## Data Availability

The datasets generated during the current study, including ultrasound movies, ultrasound measurements and high resolution images of anatomical dissections, are available from the corresponding author upon reasonable request due to their large size.

## Abbreviations

CDS: Color Doppler signal
PNC: Peripheral nerve catheter
MB: Methylene blue
